# Quantitative analysis of focal adhesion dynamics using photonic resonator outcoupler microscopy (PROM)

**DOI:** 10.1038/s41377-018-0001-5

**Published:** 2018-05-30

**Authors:** Yue Zhuo, Ji Sun Choi, Thibault Marin, Hojeong Yu, Brendan A. Harley, Brian T. Cunningham

**Affiliations:** 1 Department of Bioengineering, University of Illinois at Urbana-Champaign, Urbana, IL 61801 USA; 2 Micro and Nanotechnology Laboratory, University of Illinois at Urbana-Champaign, Urbana, IL 61801 USA; 3 Department of Chemical and Biomolecular Engineering, University of Illinois at Urbana-Champaign, Urbana, IL 61801 USA; 4 Carl R. Woese Institute for Genomic Biology, University of Illinois at Urbana-Champaign, Urbana, IL 61801 USA; 5 Atkins Building, University of Illinois Research Park, 1800 South Oak Street, Champaign, IL 61820 USA; 6 Department of Electrical and Computer Engineering, University of Illinois at Urbana-Champaign, Urbana, IL 61801 USA

## Abstract

Focal adhesions are critical cell membrane components that regulate adhesion and migration and have cluster dimensions that correlate closely with adhesion engagement and migration speed. We utilized a label-free approach for dynamic, long-term, quantitative imaging of cell–surface interactions called photonic resonator outcoupler microscopy (PROM) in which membrane-associated protein aggregates outcoupled photons from the resonant evanescent field of a photonic crystal biosensor, resulting in a highly localized reduction of the reflected light intensity. By mapping the changes in the resonant reflected peak intensity from the biosensor surface, we demonstrate the ability of PROM to detect focal adhesion dimensions. Similar spatial distributions can be observed between PROM images and fluorescence-labeled images of focal adhesion areas in dental epithelial stem cells. In particular, we demonstrate that cell–surface contacts and focal adhesion formation can be imaged by two orthogonal label-free modalities in PROM simultaneously, providing a general-purpose tool for kinetic, high axial-resolution monitoring of cell interactions with basement membranes.

## Biophotonics: imaging cell adhesion

A new imaging technique looks set to provide insights into how biological cells adhere and interact with surfaces. Yue Zhuo and coworkers from the University of Illinois at Urbana-Champaign in the USA have developed a technique called photonic resonator outcoupler microscopy (PROM). In the approach, cells to be studied are attached to the top surface of a photonic crystal biosensor, which is illuminated from below with focused light from a LED. The presence of cellular focal adhesions, a specialized protein located near the cell membrane that dictates how the cell interacts with external surfaces, serves to outcouple evanescent light from the biosensor resulting in a reduction in the sensor’s reflected signal. Measurement of this signal thus makes it possible to image focal adhesions and study their size and dynamic behavior.

## Introduction

**Fig. 1:**
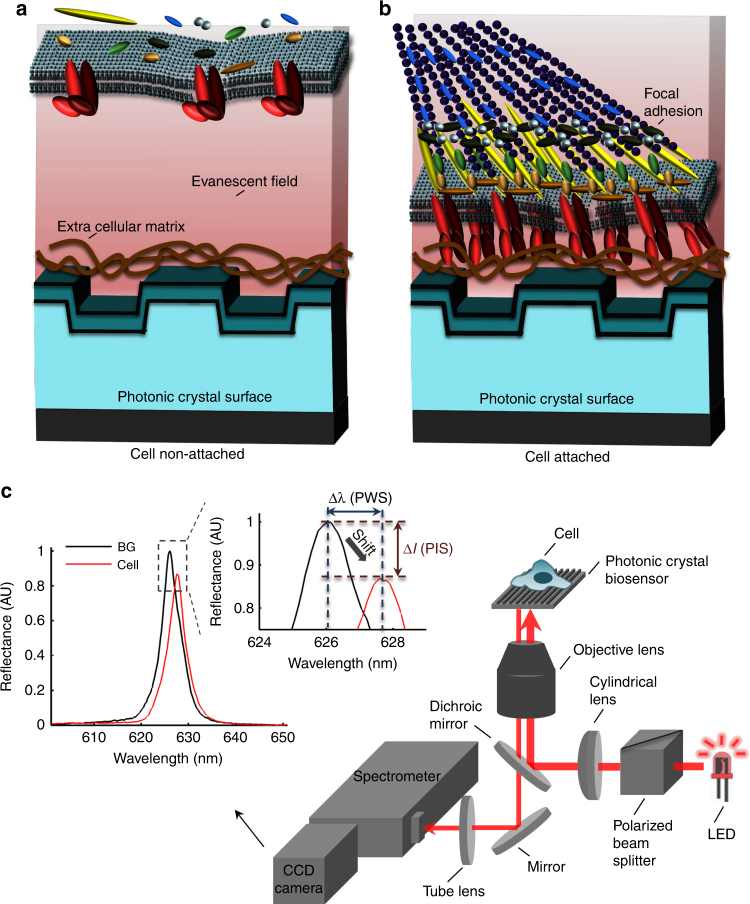
Principle of the molecular-dynamics for cell attachment on a photonic crystal (PC) biosensor in photonic resonator outcoupler microscopy (PROM). Schematic representation of the molecular mechanism **a** before and **b** after a live cell attaches to the PC biosensor surface. **c** The principle of PROM imaging system. Inset: spectra shift before and after the cell attaches to the PC surface

Focal adhesions (FAs), or cell–matrix adhesions, are large specialized proteins that are typically located at the interface between the cell membrane and extracellular matrix (ECM) (Fig. 1a, b)^1–24^. FAs are critical for supporting the cell membrane structure and regulating signal transmission between the cytoskeleton (e.g., actin) and transmembrane receptors (e.g., integrins) during adhesion and migration^16–24^. Monitoring the response of FA clusters to drugs is one important mechanism by which the action of pharmaceutical compounds may be evaluated, particularly where approaches that enable characterization to be performed with a small number of cells are especially valuable^22,25–28^. During the dynamic assembly and disassembly of a FA, the size of the FA cluster varies and is highly correlated with the level of adhesion engagement and migration speed^13,29^. For example, non-mature focal complexes (FXs) are initially formed at the leading edge of the cell (e.g., in the lamellipodia area) and are usually <0.2 µm^2^. As the lamellipodia withdraws from the leading edge, many FXs disassemble and release adhesion proteins back to the inner cell body, whereas some of the FXs grow larger (typically 1–10 µm^2^) and assemble into mature FA clusters by recruiting adapter proteins^19,29^. Once the remaining FAs are in place, they may form stationary attachment points by binding to the ECM, and a cell may utilize these anchors to migrate over the ECM by pushing and pulling the entire cellular body^18,21,23^. This insight into the dynamics of FA cluster formation and dissociation has been made possible by technical advances in the field of fluorescence and super resolution microscopy^30–36^. Optical modalities, including total internal reflection fluorescence microscopy, photoactivation localization microscopy (PALM), stochastic optical reconstruction microscopy, and interferometric PALM, coupled with fluorescence tagging of the element(s) of FA clusters via administration of fluorescently labeled antibodies or incorporation of fluorescent reporter genes by transfection of cells, along with progress made in single particle tracking algorithms, have allowed researchers to quantify FA-associated parameters, such as FA areas and sizes (*x*–*y* dimensions), FA architectures (*x*–*y*–*z* dimensions), FA turnover rates, and spatiotemporal distributions of FA complexes. Additionally, developments in traction force measurements (e.g., based on two-dimensional (2D) hydrogel substrates or micropillar substrates)^17,37–39^, mechanical probing of cells (e.g., atomic force microscopy)^40,41^, and single molecular techniques (e.g., tension sensors)^42^ have allowed the quantification of molecular tension forces within FA clusters as well as FA-mediated traction and adhesion forces.

Understanding the dynamics of FA formation and changes in FA-associated parameters is beneficial not only for understanding the fundamentals of biology but also for the field of biosensor diagnostics and screening for clinical applications^36,43,44^. Changes in FA-associated parameters, such as FA sizes and traction forces, have been linked to critical cellular processes, including metastasis, apoptosis, and chemotaxis, as well as pathologies of cancers and other diseases^9,29,36,45–47^. As such, monitoring the response of FA clusters to drugs, for example, is an important mechanism by which the action of pharmaceutical compounds may be evaluated^22,25–28,36^, and high-throughput approaches that enable the characterization of small cell populations in real time are especially valuable for these applications. Currently available techniques largely make use of fluorescence tagging to mark individual FA proteins, which entails temporal limitations imposed by photobleaching and challenges associated with accurate quantitation and long-term analysis^9,11,48^. New tools are therefore required to study the dynamic behavior of FA clusters and their interaction with the ECM to characterize changes in FA dynamics in live cells in situ. However, determining the dynamic activity of a FA cluster is challenging, especially with all of the FA proteins that are simultaneously active during the in situ assembly and disassembly processes in live cells. Although a variety of approaches have been utilized to investigate these processes, the detailed mechanism of FA assembly and disassembly in live cells, including the variability of the FA dimension, is poorly understood^9,11,48^. For instance, fluorescent tags are often used to mark individual FA proteins, but due to the temporal limitations imposed by photobleaching, accurate quantitation and long-term analysis are exceedingly difficult to perform, whereas the cytotoxicity of fluorescent tags compromises the viability of the cells under study. Here we describe a label-free optical sensing approach that combines two optical modalities to quantify the FA-associated parameters that are critical for characterizing spatiotemporal distribution patterns and the strength of FA clusters in real time. In our previous studies on cell imaging by photonic crystal enhanced microscopy (PCEM), we utilized an imaging modality in which the reflected resonant peak wavelength value (PWV) was measured over the imaging field-of-view to derive images of the peak wavelength shift (PWS) that occur when cells attach to the photonic crystal (PC) surface^49–52^. In these studies, we describe how the engagement of the cell membrane components with the surface of the PC results in highly localized shifts in the resonant reflected wavelength from the biosensor^52^, as well as the design of a modified brightfield (BF) microscope that enables visualization of cell–surface attachments with a ~0.6 × 0.6 µm^2^ pixel size. The PWS image sequences clearly show the evolution of cell attachment through the engagement of the lipid bilayer cell membrane and internal cell-associated proteins within the ~200 nm deep evanescent field region of the PC. In this study, we demonstrate a novel and orthogonal imaging modality within PCEM in which we measure the resonant reflected peak intensity value (PIV) from the PC before and after live cell attachment to acquire the peak intensity shift (PIS) at each local voxel volume (Fig. 1a, b). Images of the PIS reveal highly localized and easily observable loci of protein clusters that correlate with the spatial distribution and size of FAs observed by fluorescence microscopy. We hypothesize that the observed reduction in reflected intensity from the PC is mainly caused by the outcoupling of resonant standing wave photons via scattering.

Because this imaging modality operates using an independent sensing mechanism that obtains contrast through the formation of protein clusters at the cell–ECM interface that are capable of outcoupling light from the PC biosensor surface, we name this technique photonic resonator outcoupler microscopy (PROM) (Fig. 1c). In our previous study, we report the first observation of a reduced and highly localized reflected intensity in the context of nanoparticles with optical absorption at the resonant wavelength of the PC^50^. However, these observations were made with very high contrast and highly localized metal absorbers (for plasmonic nanoparticles) or titanium dioxide (TiO_2_) nanoparticle dielectric scatterers. Although the reduced reflected resonant intensity from the high-contrast surface-attached TiO_2_ scatterers (the refractive index of TiO_2_ nanoparticles (*n*_TiO2_ = ~2.4) is much larger than that of the surrounding water medium (*n*_2_ = ~1.333)) was the first observation with PROM, this study is the first to use PROM to observe scattered outcoupling from very low contrast FAs in live cell membranes (averaged cell *n*_cell_ = 1.35–1.38) to the surrounding medium (*n*_2_ = ~1.333). Using dental epithelial stem cells attached to a fibronectin-coated ECM surface as a representative example, we demonstrate that PWS and PIS images of the same cells display distinct and complementary information. Although the regions with the greatest PWS are at the cell–surface interface in which uniformly distributed regions with the greatest surface engagement occur, the regions with the greatest PIS represent the formation of highly concentrated protein clusters at the cell–surface interface that are capable of scattering photons. Therefore, we introduce PROM as a quantitative, dynamic, and label-free approach to observe the formation and evolution of FA cluster areas that are otherwise challenging to observe with other available imaging modalities, particularly for repeated observations of the same cell population for extended time periods.

## Materials and methods

### PC biosensor

**Fig. 2:**
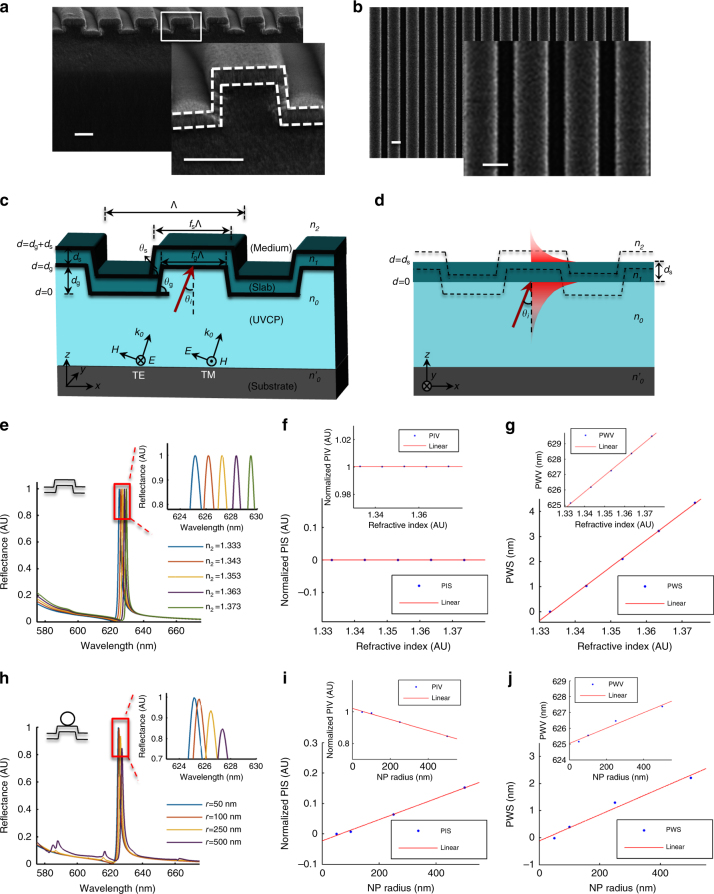
Principle of peak intensity shift (PIS) and peak wavelength shift (PWS) on a PC surface. SEM images of a fabricated PC biosensor with **a** side views of the cross-section (inset: zoomed-in side view) and **b** top views (inset: zoomed-in top view). **c** FDTD simulation model of the PC surface (side view of the cross-section). **d** Simplified model as a waveguide on the PC surface (side view of the cross-section). **e** Normalized spectra with different background refractive indices (*n*_2_ = 1.333, 1.343, 1.353, 1.363, 1.373) on a PC surface without dielectric nanoparticles (inset: zoomed-in peak of the reflection spectra). Corresponding **f** peak intensity shift (PIS) (inset: peak intensity value (PIV)) and **g** peak wavelength shift (PWS) (inset: peak wavelength value (PWV)). **h** Normalized spectra with different sizes (radius of 50, 100, 250, 500 nm) of dielectric nanoparticles on the PC surface (inset: zoomed-in peak of the reflection spectra). Corresponding **i** PIS (inset: PIV) and **j** PWS (inset: PWV). Scale bar: 200 nm

The PCs used in this study are subwavelength nanostructured surfaces with a periodic modulation in the refractive index that acts as a narrow bandwidth resonant optical reflector at one specific resonance wavelength (Fig. 1)^49,50,52–55^. The high reflection efficiency of the PC at the resonant wavelength (Fig. 1c) is the result of the formation of surface-confined electromagnetic standing waves that extend into the surrounding medium in the form of an evanescent electromagnetic field^53–80^. The photonic band gap of the PC strictly limits the lateral propagation of light. Thus the PC exhibits a strong optical confinement of incident light into an infinitesimal volume that selectively interacts with surface-adsorbed cell components while being insensitive to the components of the cell body that are not engaged with the surface. Simulations (Fig. 2) performed using the finite-difference time-domain (FDTD) method show the spatial distribution of the resonant electromagnetic field, which extends ~200 nm into the aqueous medium at the top of the PC. Previous research has demonstrated that a specific location on the PC surface has a resonant reflected wavelength that can be independently measured from neighboring regions and that the local PWV is determined by the dielectric permittivity of the biomaterial that is adsorbed at that specific location^50^. The PC surface can therefore act as a proxy for a biological surface with a built-in capacity to detect changes in the cell membrane components of cells that attach to the PC within the evanescent field, providing a compelling platform for adhesion phenotyping of single cells (see Supplementary Materials Section S-1 for details). PC biosensor surfaces are inexpensively fabricated uniformly over large surface areas by a room temperature nanoreplica molding process, as described previously in refs.^54,55^, and are incorporated onto glass microscope slides, described in refs.^49,50,52,81^.

#### Modeling the PC surface for sensor design and simulation

A numerical electromagnetics simulation package (FDTD, Lumerical Solutions, Inc., Vancouver, BC, Canada) is used to calculate the distribution of a resonant evanescent field on the PC biosensor surface. In our previous studies, the PC surface was modeled as an ideal case with a rectangular nanostructure for simplicity^50,52^. To more accurately represent the fabricated structure (Fig. 2a, b), the model used in this study incorporates a sidewall slope in a trapezoidal shape. As shown in Fig. 2c, d, the PC consists of a one-dimensional ultraviolet-curable polymer (UVCP) grating surface structure (refractive index *n*_0_ = 1.46, grating depth *d*_g_ = 120 nm, period Λ = 400 nm, duty cycle *f*_g_ = 41.6%, sidewall angle *θ*_g_ = 85°) coated with a thin film of TiO_2_ (refractive index *n*_1_ = 2.4, slab thickness *d*_s_ = 61 nm, duty cycle *f*_s_ = 50%, sidewall angle *θ*_s_ = 82°) to generate a resonant reflected narrowband mode at a wavelength near *λ*_0_ = ~626 nm. The adhesion of FAs on the PC surface is also modeled in FDTD, where the FA is represented as a homogeneous and lossless sphere (*n*_FA_ = ~1.46, radius range 50–500 nm) composed of many protein molecules (Fig. 2e–j).

#### Fabrication and preparation of the PC surface

A room temperature replica molding approach is used to fabricate the PC on a glass substrate using a quartz mold template with a negative volume image of the desired grating structure (fabricated with e-beam lithography and reactive ion etching). First, the quartz mold template is thoroughly cleaned with a piranha solution (a mixture of sulfuric acid (H_2_SO_4_) and hydrogen peroxide (H_2_O_2_), H_2_SO_4_:H_2_O_2_ = 3:1) for approximately 3 hours to remove organic residues from the surface of the master template. The glass substrate is cleaned in an ultrasonic bath three times with isopropyl alcohol (IPA), acetone and deionized (DI) water for 1 min in each solvent and then dried with nitrogen gas and treated with oxygen plasma. Second, the liquid UVCP is deposited between the quartz mold template and glass substrate, and a high intensity UV lamp is used to cure the liquid polymer to a solid state. After peeling the grating replica away from the quartz mold template, the nano-patterned surface is attached to a glass cover slip with an adhesive. Then PC fabrication is completed by reactive sputter deposition (PVD 75, Kurt J. Lesker, Jefferson Hills, PA, USA) of a high refractive index thin film (TiO_2_) atop the grating structure. Scanning electron microscopic (SEM) images of a cross-sectional view and a top view of the structure are shown in Fig. 2a, b, respectively. Next, before cell attachment experiments, the PC is cleaned in an ultrasonic bath with IPA and DI water for 1 min each, followed by drying with nitrogen gas. The PC is then treated with oxygen plasma to facilitate attachment of a liquid containment gasket formed from polydimethylsiloxane. Finally, the PC surface is hydrated with a phosphate-buffered saline solution and coated with a layer of ECM molecules (e.g., fibronectin) to promote cellular attachment.

### Photonic resonator outcoupler microscopy

The PROM instrument is a modified BF microscope that uses a line-scanning approach to measure the spatial distribution of optical spectra across a PC surface with a submicron spatial resolution in the axial direction for label-free imaging (Fig. 1c)^49,51^. An optical fiber-coupled light-emitting diode is used as the light source, and a line-profiled (polarized perpendicular to the grating structure) light beam illuminates the PC biosensor from below through a microscope objective lens (e.g., 10×). Illumination from below eliminates the possibility of the scattering, absorption, and meniscus reflection and refraction of materials in the cell media or cell body from effecting a resonant reflected signal to the PC surface. The reflected light, containing the resonant reflected spectrum, passes through the objective lens in the opposite direction and through the entrance slit of an imaging spectrometer and is finally collected by a charge-coupled device camera, which records the resonant reflected spectrum from each pixel across the illuminated line on the PC surface. A high spatial resolution in the axial direction is obtained due to the shallow evanescent field of the PC (~200 nm). The resolution in the lateral direction is determined by the lateral propagation distance of resonant-coupled photons, resulting in the detection of distinct surface-attached objects for widely dispersed features at the micron size scale^50^. The same field of view can be re-scanned repeatedly to generate a sequence of images for the same cells. The current shortest available scan interval for our instrument is ~10 s for ~100 × 100 µm^2^, which is limited by the exposure time and speed of the motorized scan stage. While characterizing the resolution performance through the intentional introduction of dielectric and metallic nanoparticles of a variety of sizes (30–500 nm), we observe that dielectric objects not only induce a shift in the PWV but also a reduction in the resonant peak intensity^50^. When a cell attaches to the PC surface, the peak resonant wavelength red-shifts from a lower wavelength (before cell attachment, e.g., *λ*_BG_ = ~626 nm) to a higher wavelength (after cell attachment, e.g., *λ*_cell_ = ~628 nm). At the same time, the resonant reflection efficiency, as measured by the peak intensity, changes from a higher PIV (before cell attachment, e.g., ~90% normalized to a peak reflectance of 100% for the PC immersed in water) to a lower value (after cell attachment, e.g., 80% as a normalized PIV). A negative PIV shift indicates that proteins in some areas of the cell bind to transmembrane proteins (e.g., integrins) to form more substantial FA clusters. The PROM instrument and sensor structure measure the resonant reflection characteristics of the PC via the spectrum obtained from each ~0.6 × 0.6 µm^2^ pixel area, representing a total field of view region (e.g., ~300 × 300 µm^2^) of the PC surface.

### Stem cell culture

Murine dental epithelial stem cells (mHAT9a) were maintained in Dulbecco’s Modified Eagle Medium supplemented with 10% fetal bovine serum and 1% Penicillin–Streptomycin. Stem cells were cultured at a temperature of 37 °C and supplied with an environment of 5% CO_2_ humidified air during imaging.

## Results and Discussion

### Electromagnetic computer modeling of resonant outcoupling from a PC by FA

It is important to understand the physical mechanism that is responsible for the PIS in the context of cell attachment. Theoretical and experimental analyses suggest that a reduction in the PIV can occur by two mechanisms: (1) materials that act as efficient absorbers of the resonant wavelength (such as gold nanoparticles) locally quench the PC resonance; (2) concentrated local regions of high dielectric permittivity that can outcouple resonantly confined light by scattering^50^. Interestingly, by analyzing PROM data during cell attachment, we observed the characteristics of PIV images that differ substantially from PWS images. Although optical absorption at the PC resonant wavelength will efficiently reduce the PIV in a highly localized manner, the protein and lipid components of cells and cell membranes do not display strong absorption in the visible wavelength range. Human tissues and live cells exhibit strong absorption in the infrared wavelength range; however, these wavelengths are not utilized in our detection approach and thus are unlikely to be the dominant factors that contribute to PIV reduction. Additionally, though metallic elements comprise a small fraction of a cell’s atomic constituency, metal atoms are present as ions rather than as clusters that are capable of optical absorption in the visible part of the spectrum. Scattering occurs when light is forced to deviate from its original trajectory due to localized non-uniformities in its propagation medium, which occur, for example, when light propagating through water is reflected or refracted by a particle with a greater refractive index. A highly concentrated region (e.g., FA cluster) with a greater refractive index than its surroundings (e.g., cell media) generates more localized and efficient scattering than a diffuse region with a gradual gradient in the refractive index. Scattering effects also become stronger when the size of a region with a refractive index contrast increases. Because the cross-sectional area of a FA cluster is typically 0.2–1.0 µm^2^, we can expect to observe measurable differences in the scattering efficiency of membrane-associated protein clusters as they form, change size, and subsequently dissipate. Light scattering from internal cell components, such as organelles and mitochondria, has recently been utilized to achieve imaging contrast in the context of changes that occur in precancerous cells that express phenotypic changes due to the expression of mutant genes^82–84^. In PROM, we detect the modulation of membrane-associated scattering that occurs due to FA formation by utilizing the ability of localized high refractive index protein clusters to produce image contrast by reducing the reflection efficiency of a PC biosensor.

After analyzing the mechanism of PIV reduction, we study the useful cellular information that can be uniquely extracted from the measurement of this physical quantity. Our hypothesis is that the dominant cause for the measured PIV reduction is light scattering rather than absorption. Thus a scattering model can represent the interaction of light with a FA cluster since scattering describes the effect of an electromagnetic plane wave propagating through a dielectric particle. To predict the effects on the resonant reflection spectrum from a PC that can be induced by a FA on its surface, we compared the computed reflection spectra with and without a small region of dielectric contrast on a PC surface. In a FDTD electromagnetic computer model of a FA on a PC surface (Fig. 2e–j), we represent the FA as a locus of a material with a designated radius (50–500 nm) and designated refractive index elevation (*n*_FA_ = ~1.46) in contrast to the surrounding medium (estimated to be *n*_2_ = ~1.333). The simulation results demonstrate that, when the FA cluster is *not* present, the PWS in the resonant reflection spectrum increases (Fig. 2g) as the refractive index contrast of the surrounding media increases, whereas the PIS remains the same (Fig. 2f). However, when the FA cluster is present, as the radius of the FA increases (Fig. 2h), the maximum in the resonant reflection spectrum decreases (Fig. 2i, Inset), indicating that more energy is outcoupled from the PC resonant standing wave. Thus the PWSs to a higher wavelength (Fig. 2j), as expected, and the PIS (compared with the original PIS without a FA cluster) increases as the FA cluster size increases (Fig. 2i) due to the scattering-induced outcoupling (for more details, see Supplementary Materials S-2).

### Dynamic PIS images of live cells

**Fig. 3:**
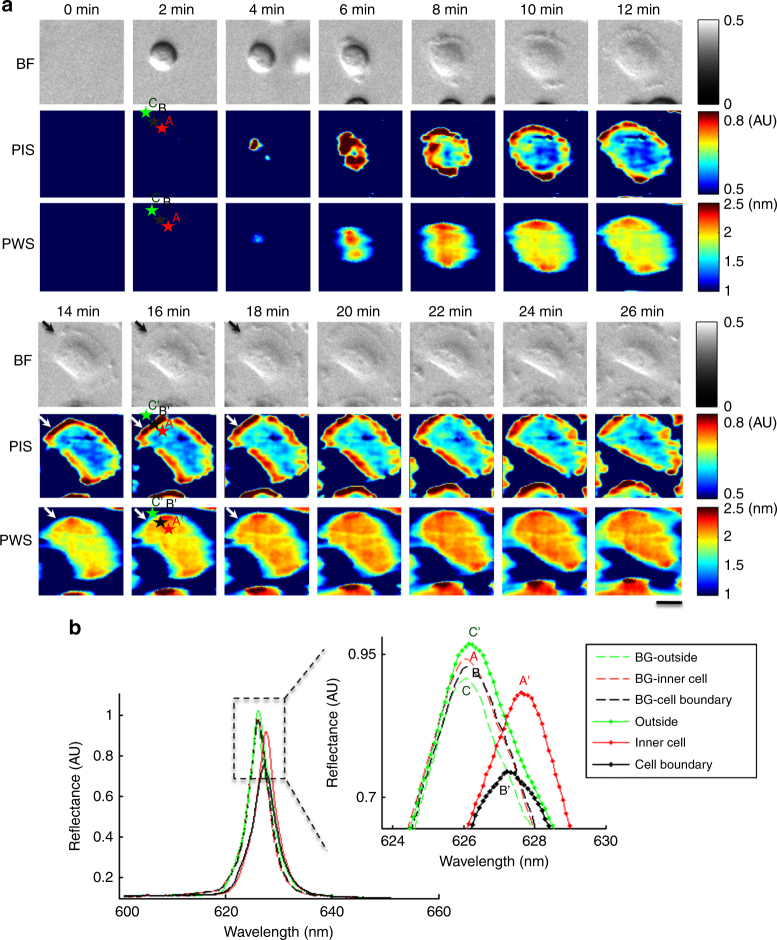
PIV images and spectra during live stem cell (mHAT) attachment. **a** Sequence of PROM-acquired images at several time points (0–26 min) during the cell adhesion process. Top row: brightfield (BF) images; middle row: peak intensity shift (PIS) images; bottom row: peak wavelength shift (PWS) images. **b** PROM-measured spectra at three locations on the PC surface before (A, B, C, in dashed lines) and after (A’, B’, C’, in dotted lines) cell adhesion. Point A’ (red) represents the inner cell area; point B’ (black) represents the cell boundary; point C’ (green) represents the outside of the cell boundary. Scale bar: 20 μm

Dynamic PIS images can be acquired by PROM during live cell adhesion over an extended time period to create movies of FA development, which are difficult to acquire via fluorescence imaging due to photobleaching. The PIS here is defined such that greater reductions in the reflection efficiency are displayed as higher intensity values for a simpler visual comparison with other imaging modalities. As shown in Fig. 3a, the resulting PIS image sequence reveals that the cell periphery has a greater degree of scattering than the cell center. This “ring” effect demonstrates that the PIS intensity is not homogeneously distributed throughout the cell membrane. Fig. 3b shows spectra from three sample points marked in Fig. 3a (A—red, B—black, C—green) at different times. Initially, all three points are located outside of the cell (~0 min), and all of the points show high resonant PIVs as highlighted by the dashed line in the spectra (Fig. 3b). During cell adhesion, the attachment perimeter expands and surpasses the three points as the cell extends its attachment area. Once the cell firmly attaches and adheres to the PC surface (~16 min), points A’, B’, and C’ represent locations inside, near, and outside of the cell boundary, respectively. The solid lines in Fig. 3b demonstrate that the spectra of point A’ shifts to a lower resonance peak intensity, point B’ shifts to the lowest resonance peak intensity, and point C’ remains at the original resonance peak intensity. Considering all of the pixels within the cell, we observe a ring of enhanced scattering that encompasses much of the cell periphery.

**Fig. 4:**
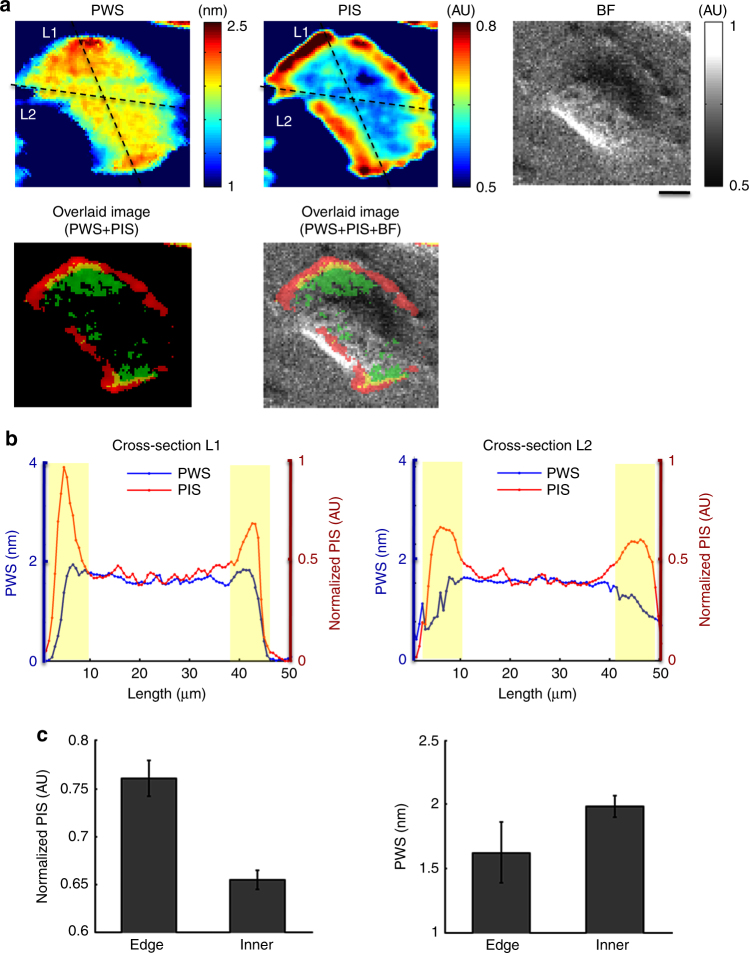
Zoomed-in PROM images and cross-section (before and after mHAT cell adhesion). **a** PROM-acquired images (including PWS, PIS, and BF images) and their overlap images at ~16 min. **b** Comparison of cross-sectional curves (L1 and L2) of the PWS and PIS images. **c** Bar graph of the statistical comparison between the edge and the center of the cells. Scale bar: 20 μm

A difference of PWS and PIS images for the same cell at each time point is clearly observed in the spectral data acquired by PROM. In Fig. 3b, the dashed lines represent the background spectra for a representative pixel acquired before cell attachment (0 min), and the dotted lines with dot markers represent the spectra of the same pixel after cell attachment (~16 min). PWS and PIS images are simultaneously extracted from the spectra data at the PWV and PIV, respectively (at every ~0.6 × 0.6 µm^2^ pixel area on the PC surface). In Fig. 3a, the regions with the greatest values in the PWS and PIS images show distinct distribution patterns, which suggests that these regions may represent two different physical mechanisms. For instance, the PWS image of the cell marked by the white arrow (e.g., ~16 min) has a high PWS on the top and bottom of the cell body, whereas the PIS image of the same cell has a “ring” of a high PIS along the cell boundary. The zoomed-in images of the PWS and PIS taken 16 min after introduction of a single cell are shown in Fig. 4a and S-Fig. 1, respectively, and the overlaid PWS and PIS images (high intensity values only, PWS—green, PIS—red, overlap—yellow) over the BF image of the same cells show the distinct distribution patterns between these two physical quantities. The cross-sectional curves (L1 and L2) along the diameter of the cell are plotted in Fig. 4b, and the corresponding statistical results are shown in Fig. 4c (*N* = 5 cells). The high intensity of the PWS (in green at the bottom of Fig. 4a) represents a higher mass density of cellular materials associated with the cell membrane. The high intensity of the PIS (in red at the bottom of Fig. 4a) along the cell boundary represents the scattering outcoupling effect from the locally generated FA clusters. Typically, a larger cluster size of protein aggregates corresponds to a greater PIV reduction compared with the background. Therefore, these measurements of local PWS and PIS can quantify the surface-attached cellular mass density and dimension of the FA clusters dynamically and simultaneously.

### Comparison of PIS and fluorescence images

**Fig. 5:**
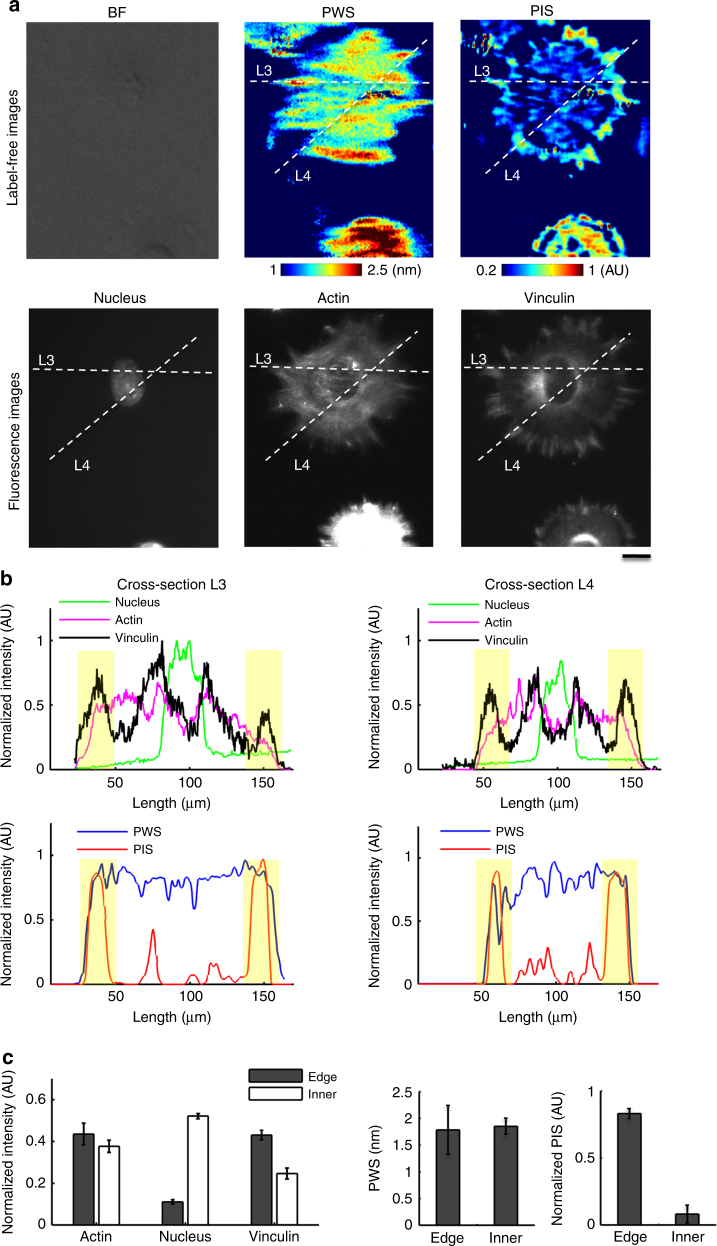
Comparison of label-free images and fluorescence images with cross-section. **a** Top row: brightfield (BF), PWS, and PIS images; bottom row: fluorescence images, including dyes that selectively stain the nucleus, actin, and vinculin. **b** Comparison of the image cross-sections through lines (L3 and L4) for the fluorescent (nucleus—green, actin—magenta, vinculin—black), PWS (blue), and PIS (red) images. Light-yellow regions represent the regions near the cell edges, where the vinculin (black curves) and PIS (red curves) both have high intensities. **c** Statistical comparison among the fluorescence images (actin, nucleus, and vinculin) and label-free images (normalized PWS and PIS value comparison between cell edges and centers). Scale bar: 20 μm

Fluorescence images were acquired for the same cells to further investigate the FA areas detected in the PIS images. In Fig. 5a, the top row shows BF, PWS, and PIS images and the bottom row displays fluorescence images of the same cell with fluorescence tags applied to three different cellular components (nucleus, actin, and vinculin). There is no obvious pattern similarity between the PIS image and actin (indicating the presence of cytoskeleton components) or nucleus images. However, the fluorescent image of vinculin (a type of FA molecule) in the bottom row of Fig. 5a indicates that filopodia reside in the FA area along the stem cell boundary. As shown in Fig. 5a in the right column, the PIS image shows a nearly identical distribution pattern along the cell peripheral region to that obtained by fluorescence microscopy with a labeled vinculin where the FA areas are concentrated along the cell boundary. Fig. 5b shows two cross-sections along different radial directions (L3 and L4, as shown in Fig. 5a) sampled across the cell diameter for the PWS (blue curves), PIS (red curves), and fluorescence-tagged images (nucleus—green, actin—magenta, vinculin—black). Both the PIS (in red) and vinculin (in black) curves exhibit a similar “ring” effect along the cellular boundary (highlighted in the light-yellow regions), which indicates that the high intensity in the PIS images are probably co-localized with the FA areas. Therefore, the dimensional change of the FA cluster can also be detected with PIS images using PROM.

Statistical analyses are shown in Fig. 5c (*N* = 5 cells) for the fluorescent, PIS, and PWS images along the cell boundary (marked as “Edge”) and within the nucleus area (marked as “Inner”). Fig. 5c (Left) displays fluorescence images (including actin, nucleus, and vinculin) that demonstrate three different patterns of distribution along the cell edge and center. The nucleus image only shows high intensity within the area of the nucleus because the fluorescent molecular probes only tag nucleic acid material, such as chromosomes. Actin mainly functions as a cytoskeleton molecule that is rapidly remodeled by dynamically forming microfilaments to support the cell structure or participating in many important cellular processes, including cell division or cell signaling. As a scaffold protein, the distribution of actin is relatively uniform, and thus the difference of the fluorescence intensity between the cell edge and center in the actin image is small. Vinculin is a membrane-cytoskeletal protein that is often localized in the FA area because it participates in the linkage between the transmembrane protein (e.g., integrin) and cytoskeletal protein (e.g., actin). Therefore, a fluorescence dye for vinculin is often used to visualize the locations of FAs. Fig. 5c clearly shows that the distribution of vinculin is mainly along the cell periphery for a surface-attached cell, which is highly consistent with the distribution pattern of the PIS (high in “Edge”, low in “Inner”) (Fig. 5c Right), which is not similar to that of the PWS (Fig. 5c Middle). There is a small difference between the PIS image and vinculin fluorescence image around (outside of) the nucleus. This is likely because the vinculin fluorescence image is a transmission image in the axial direction across the entire cell body (with a thickness of several microns to several tens of microns) including the nucleus area. By contrast, the PIS image is only measured through the evanescent field, which has a thickness that is several hundred times thinner (several tens of nanometers) in the axial direction starting from the bottom of the cell body (before reaching nucleus).

**Fig. 6:**
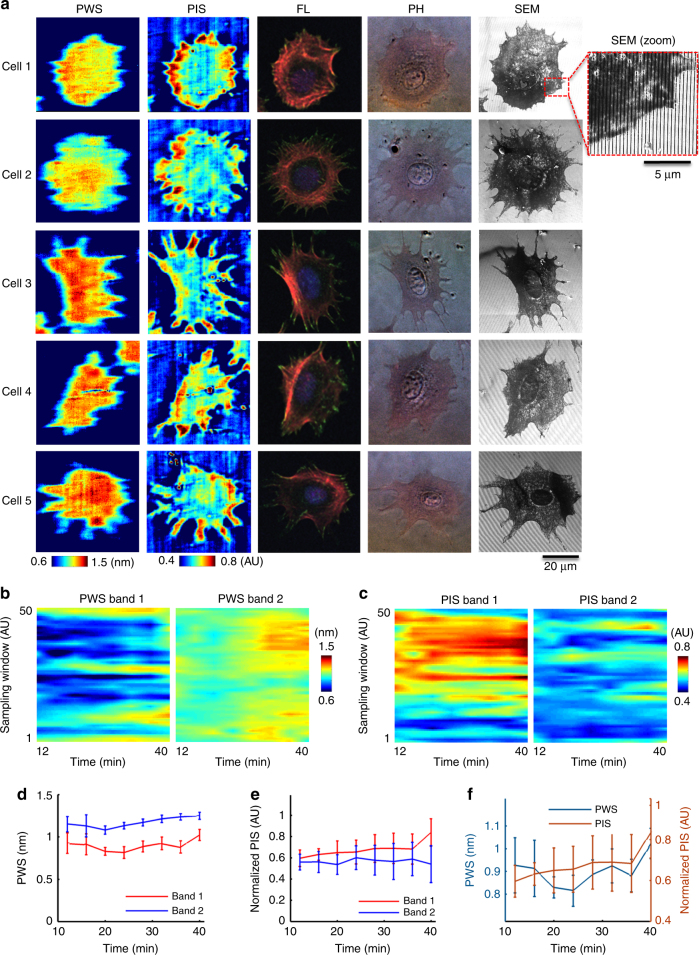
Image modality comparison and dynamic analysis of PROM images during cell adhesion. **a** Five selected cells imaged by peak wavelength shift (PWS), peak intensity shift (PIS), confocal fluorescence microscopy (FL) (red—actin, green—vinculin, blue—nucleus), phase-contrast microscopy (PH), and scanning electron microscopy (SEM). Scale bar: 20 μm. (Top-right inset: zoom-in SEM image for cell 1. Scale bar: 5 μm). 2D spatiotemporal maps are tracked from different bands (band 1—near cell boundary, band 2—inner region of cell) in stem cells with **b** PWS images and **c** normalized PIS images. Mean and standard deviation of the temporal curves of cell adhesion for **d** PWS, **e** normalized PIS, and **f** the comparison between PWS and normalized PIS

To highlight how information from PROM images complements those obtained by orthogonal imaging modalities, five selected stem cells are shown in Fig. 6a imaged by PWS images, PIS images, confocal images with fluorescence dyes (FL), phase-contrast images (PH), and SEM images. PROM images obtained using PWS and PIS information reveal different features of cell attachment, and both show clear details of the cell attachment boundary. PWS and PIS images highlight only behavior associated with the cell–ECM interface and thus do not show material in the upper cell body. Unlike SEM and fluorescence images, PWS and PIS images yield dynamic and highly quantitative information that can be visualized graphically. As shown in Fig. 6b, c, the stem cell boundary can be tracked along the local normal direction frame-by-frame, enabling the PIS to be sampled along the cell boundary spatially and temporally at the same time. Associated dynamic analyses with different sampling bands (S-Fig. 2, black for band 1—near cell boundary, white for band 2—inner region of the cell) in cells were performed on PWS images and PIS images. The resulting 2D maps shown in Fig. 6b, c represent the PWS and PIS with spatiotemporal information along the cell boundary and time frames. Comparing the different bands between both maps, the PIS increase is much higher in band 1 compared with that in band 2 (Fig. 6c), whereas the PWS shows the opposite trend (Fig. 6b). The dramatic increase of the PIS may be due to the aggregations of FAs along the cell boundaries, which is confirmed in the FL images (Fig. 6a). The mechanisms of the temporal curves for cell adhesion for the PWS and PIS are shown in Fig. 6d, e (*N* = 5 cells). Directly comparing the dynamics between the PWS and PIS is difficult because they have different dynamic ranges. However, different slopes indicating different increasing ratios along the temporal dimension are observed if the PWS and normalized PIS are plotted together, as shown in Fig. 6f.

The PROM images show cell borders and intra-cell features that are approximately in the order of the pixel size limit in the axial direction, which is near the diffraction limit of the resonant wavelength. It is important to put these images in the context of PCEM images that were gathered previously from high-contrast objects. In the lateral directions, although the effect of a point dielectric object on the reflected wavelength from a PC can extend to the surrounding pixels (because the electric field standing wave “samples” a greater lateral dimension than one period), the outcoupling from a surface adsorbed scatterer (or absorber) is observed to be more highly localized. The full-widths at half-maximums of the point spread function of TiO_2_ nanoparticles (e.g., diameter of ~100 nm) were measured as ~1.20–1.56 µm for PWS images and as ~0.95 µm for PIS images^50^. Our previous study shows that the spatial resolution of the dielectric objects in PWV-based PCEM images is directly correlated with the refractive index contrast of the object. Although high-contrast objects (such as a TiO_2_ dielectric nanoparticle or the edge of a photoresist pattern) can extend their “influence” on the measured PWV by as much as a couple of microns (e.g., 2–3 μm) in any direction, lower-contrast objects have a much more limited perturbation. In PROM images of attached cells, there is an extremely low refractive index contrast between the attached cell membrane and surrounding cell media. Within the footprint of an attached cell, the refractive index contrast between a FA and the neighboring cell membrane regions is even lower. These hypotheses were formed by the contrast observed at the cell attachment border and the contrasting regions of attachment within a cell. Instead of observing smeared borders that extend for several microns, we observed a contrast in PIS and PWS images with micron-scale features. These observations are consistent with our earlier measurements but have been applied here for the first time in the context of attached cell images of the PIS.

## Conclusions

This study describes a label-free microscopic approach that quantitatively measures the scatter-induced changes in the reflected intensity from a PC biosensor surface to reveal the kinetic evolution and spatial features of FAs that form at the cell–surface interface. Compared to a sensing approach in which image contrast is generated by the dielectric permittivity of attached cell components, PROM provides contrast in the reflected resonant intensity that is induced by the refractive index contrast of the localized protein clusters that occur at the cell–surface interface, which comprise FA sites. Our hypothesis is supported by electromagnetic computer simulations that have modeled small and low refractive index contrast regions on a PC that induce measurable reductions in the resonant reflection efficiency. Our hypothesis is also supported by fluorescence microscopy of cells in which the patterns of FA regions are similarly distributed as patterns of reflected intensity reduction measured by PROM. We show that images of the PIS and PWS can be gathered from the same spectral information for the same cells and that the two imaging modalities have distinct spatial patterns and thus provide complementary information about cell–surface activity. Dynamic images of the PIS and PWS can be repeatedly gathered over extended time periods with a 10-s temporal resolution via a line-scanning approach to generate time-course movies of cell–surface behavior during processes that occur over several hours. As a label-free imaging approach, PROM does not suffer from the limitations of fluorescence-based microscopy, which include photobleaching and stain cytotoxicity. We expect PROM to be a highly useful tool that can reveal the mechanisms of biological processes that occur near the cell membrane when the membrane is attached to ECM materials during cell migration, division, metastasis, apoptosis, and stem cell differentiation.

## Electronic supplementary material

Supplementary Figures(PDF 483 kb)

Supplementary Materials(PDF 344 kb)
