# Supplementary material for: Quantitative analysis of focal adhesion dynamics using photonic resonator outcoupler microscopy (PROM)
Source: Light Sci Appl. 2018 May 30;7:9. doi: 10.1038/s41377-018-0001-5 (PMC6020849; doi:10.1038/s41377-018-0001-5)
Supplement: Supplementary file 1 — Supplementary Figures(PDF 483 kb) [file 41377_2018_1_MOESM1_ESM.pdf]

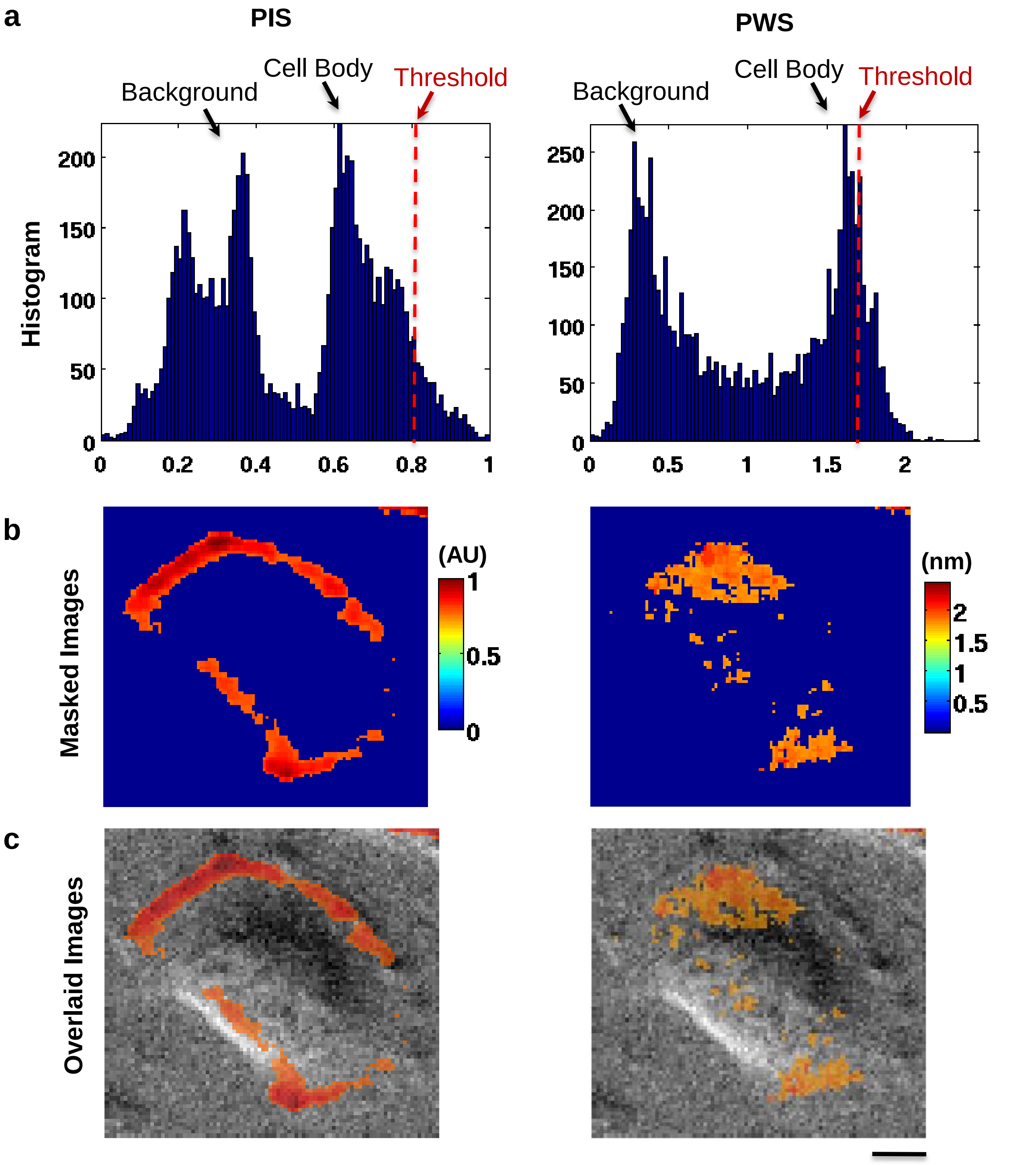


**S-Fig. 1. Mask with selected threshold for image overlay.** (a) Threshold selection from the PIS and PWS histogram is used to reveal locations with the greatest cell attachment "strength" as measured by PWS and locations with the greatest outcoupled scattering, representing focal adhesion sites, as measured by PIS. (b) Masked high intensity of PIS and PWS images. (c) Overlaid PIS and PWS images with brightfield images of the same cell. Scale bar: 20 μm.

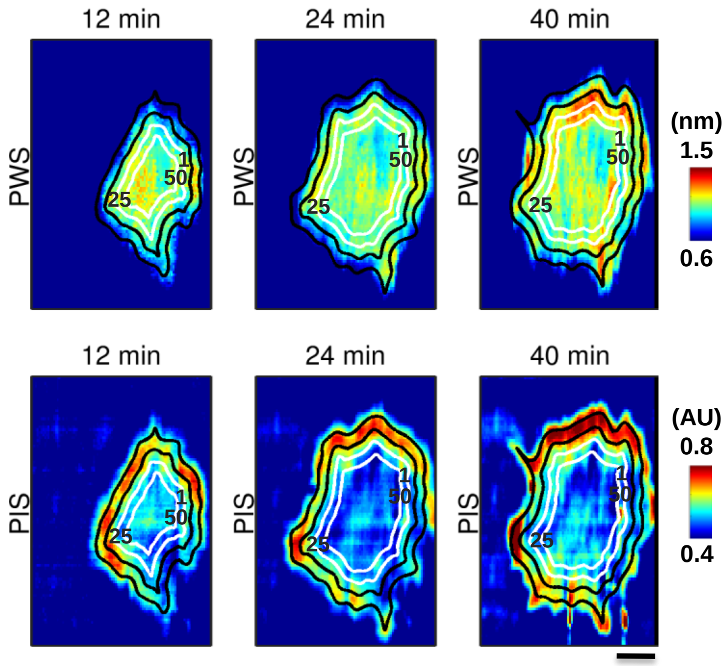


**S-Fig. 2. Different locations of the cells in different time frames.** Selected different bands at different locations in cells for the PWS (first row) and normalized PIS (second row) images at different times (12, 24, and 40 minutes) after seeding the cells on a photonic crystal surface. Black curves represent band 1 (near the cell boundary), and white curves represent band 2 (inner region of the cell). Scale bar: 20 µm.
