# Supplementary material for: Quantitative analysis of focal adhesion dynamics using photonic resonator outcoupler microscopy (PROM)
Source: Light Sci Appl. 2018 May 30;7:9. doi: 10.1038/s41377-018-0001-5 (PMC6020849; doi:10.1038/s41377-018-0001-5)
Supplement: Supplementary file 2 — Supplementary Materials(PDF 344 kb) [file 41377_2018_1_MOESM2_ESM.pdf]

Supplementary Materials

Yue Zhuo[1,4], Ji Sun Choi[2,5], Thibault Marin[6], Hojeong Yu[3], Brendan A. Harley[2,5], Brian T. Cunningham[1,3,4,5*]

[1]Department of Bioengineering, [2]Department of Chemical and Biomolecular Engineering; [3]Department of Electrical and Computer Engineering, [4]Micro and Nanotechnology Laboratory, [5]Carl R. Woese Institute for Genomic Biology, University of Illinois at Urbana-Champaign, Urbana, Illinois 61801, USA
[6]Atkins Building, University of Illinois Research Park, 1800 South Oak Street, Champaign, IL 61820, USA
*E-mail: bcunning@illinois.edu

S-1 The physics of the PC surface

A photonic crystal (PC) is a periodic arranged nanostructure of the high and low refractive indices materials satisfying moment-matching conditions in certain incident direction within certain wavelength range (Fig. 2a for side view, Fig. 2b for top view)[49-83]. To understand the physics of such complex nanostructure, the PC can be modeled as more simplified nanostructures based on different purposes. Typically, when the light is confined in an optical cavity (e.g., between a pair of parallel mirrors face-to-face, or between two faces of a high refractive index slab), it reflects multiple times within the cavity and forms a standing wave when a specific resonant wavelength matches with the cavity length. The PC nanostructure (Fig. 2c) can be approximated as an asymmetric dielectric slab waveguide (Fig. 2d) (since the dielectric constant in the substrate is different with that of the medium above the slab)[64-67,77-80]. Based on the temporal coupled mode theory (CMT), the fields on the PC surface can be described with the Helmholtz wave equation,

$$\left(\nabla^2 + \boldsymbol{k^2}\right)\mathbf{E} = 0, \tag{1}$$

where ∇^2 is the Laplace operator; k is the wavenumber for the wave vector $\boldsymbol{k}$ and $k = \omega^2\mu\varepsilon$ (where ω, μ, ε are angular frequency, permeability, and permittivity, respectively); **E** is the electric field in the structure. Here the polarization of the incident radiation is defined as follows: 1) the transverse magnetic (TM) modes represent that the magnetic field (**H**-field) vector is parallel to the grating; 2) the transverse electric (TE) modes represent that the electric field (**E**-field) vector is parallel to the grating. The field solutions can be estimated for the fields in both TM and TE polarizations. In TM polarizations, the magnetic fields of first-order diffracted wave can be written as $\mathbf{H} = \hat{y}H_y$, where

$$H_y = \begin{cases} Ae^{\alpha_0 z}e^{-ik_x x}, & z \le 0 \\ \left(Be^{-ik_{1z}z} + Ce^{ik_{1z}z}\right)e^{-ik_x x}, & 0 \le z \le d_s \\ De^{-\alpha_2 z}e^{-ik_x x}, & z \ge d_s \end{cases} \tag{2}$$

Where H_y is the y-component of magnetic field **H**; α_0 and α_2 are the decay constants of the evanescent fields in the substrate under the slab and background medium above the slab, respectively; A, B, C, D are the constants for the magnetic fields; d_s is the slab thickness as shown in Fig. 2d. Similarly, in TE polarizations, the electric fields of first-order diffracted wave can be written as $\mathbf{E} = \hat{y}E_y$, where

$$E_{\boldsymbol{y}} = \begin{cases} \frac{\alpha_0}{n_0^2}\frac{i}{\omega\varepsilon_0}Ae^{\alpha_0 z}e^{-ik_x x}, & z \le 0 \\ -i\frac{k_{1z}}{n_1^2}\frac{i}{\omega\varepsilon_0}\left(Be^{-ik_{1z}z} - Ce^{ik_{1z}z}\right)e^{-ik_x x}, & 0 \le z \le d_s \\ -\frac{\alpha_2}{n_2^2}\frac{i}{\omega\varepsilon_0}De^{-\alpha_2 z}e^{-ik_x x}, & z \ge d_s \end{cases} \tag{3}$$

Where E_y is the y-component of electric field **E**; n_0, n_1, and n_2 are the refractive indices in the substrate under the slab, the slab, and the background medium above the slab, respectively (each type of medium has been assumed to have uniform permittivity and permeability); ε_0 is the permeability of free space.

S-1.1 Momentum-matching condition

Along x direction in the nanostructure (Fig. 2c), the PC slab can be viewed as a high refractive index diffraction grating with a photonic band-gap. When total internal reflections occur in the PC slab, the momentum-matching condition can be used to describe the Bragg scattering in the grating (which only allows the 0th- and 1st-order diffraction here),

$$k_{1x}^{(m)} = k_{0x} \pm mG_x \tag{4}$$

where the $k_{jx}^{(m)}$ is the x-component of the wavenumber in the jth region of materials (j = 0, 1, 2; 0th-substrate, 1st-slab, 2nd-background medium), and m is the order of the diffraction; k_{0x} is the x-component of the wavenumber of the incident light in the substrate under the slab and $k_{0x} = |\boldsymbol{k_0}|\sin\theta_i$(where θ_i is the incident angle, $\boldsymbol{k_0}$ is the wave vector in the substrate); G_x is the reciprocal lattice vector of the grating and $|G_x| = \frac{2\pi}{\Lambda}$ (where Λ is the period of the nano grating). When the resonance condition is satisfied, the incident light at the resonant wavelength can couple into the in-plane guided resonance modes in the PC slab.

S-1.2 Guided resonance (GR) mode

For the guided resonance modes in the slab, the wavenumber (k_{1z}) along z direction can be described by the dispersion relation

$$\begin{cases} k_0^2 = k_x^2 - \alpha_0^2, & z \le 0 \\ k_1^2 = k_x^2 + k_{1z}^2, & 0 \le z \le d_s \\ k_2^2 = k_x^2 - \alpha_2^2, & z \ge d_s \end{cases} \tag{5}$$

where k_j is the wavenumber in the jth region of materials (j = 0, 1, 2); k_x is the wavenumber along x-direction and is the same in each layer of material when satisfying the phase matching condition. Boundary conditions at the region 0-1 interface (substrate/slab) and region 1-2 interface (slab/medium) can be used to solve for the guidance conditions,

$$\begin{cases} H_{0y} = H_{1y}, \quad E_{0y} = E_{1y}, & z = 0 \\ H_{1y} = H_{2y}, \quad E_{1y} = E_{2y}, & z = d_s \end{cases} \tag{6}$$

with both TM and TE polarizations. Where H_{jy} and E_{jy} are the y-components of magnetic and electric fields in the jth region of materials, respectively. From Equations (2), (3), (5) and (6), the guidance condition can be found to be

$$\sqrt{(k_0 n_1)^2 - k_x^2}\, d_s = \tan^{-1}\left(\frac{n_1^2}{n_0^2}\frac{\alpha_0}{\sqrt{(k_0 n_1)^2 - k_x^2}}\right) + \tan^{-1}\left(\frac{n_1^2}{n_2^2}\frac{\alpha_2}{\sqrt{(k_0 n_1)^2 - k_x^2}}\right) + p\pi \tag{7}$$

for the pth order of TM modes, and

$$\sqrt{(k_0 n_1)^2 - k_x^2}\, d_s = \tan^{-1}\left(\frac{\alpha_0}{\sqrt{(k_0 n_1)^2 - k_x^2}}\right) + \tan^{-1}\left(\frac{\alpha_2}{\sqrt{(k_0 n_1)^2 - k_x^2}}\right) + p\pi \tag{8}$$

for the pth order of TE modes.

S-1.3 Reflection of Fabry-Perot (FP) cavity

Along z direction, a Fabry-Perot cavity can exist because the high refractive index material (e.g. TiO_2) has a finite thickness in submicron regime, which can match with certain wavelength in the visible light range. Therefore, the thickness of the TiO_2 determines simultaneously the continuum broadband FP reflection and the discrete narrowband guided mode dispersion. The vertical FP reflection can be estimated by approximating the nano structure as a thin film without the grating. The incident, reflected, and transmitted electric fields hence can be put in a form with propagation matrix approach,

$$\begin{bmatrix} E_0 \\ r_f E_0 \end{bmatrix} = \boldsymbol{B} \begin{bmatrix} t_f E_0 \\ 0 \end{bmatrix} \tag{9}$$

where t_f and r_f are the transmission and reflection amplitudes for the vertical FP cavity, respectively; E_0 is the amplitude of the electric field of incident light; $\boldsymbol{B}$ is the backward propagation matrix and can be described as

$$\boldsymbol{B} = \boldsymbol{B_1}\boldsymbol{B_0} = \begin{bmatrix} b_{11} & b_{12} \\ b_{21} & b_{22} \end{bmatrix}, \tag{10}$$

when the incident light is normal to the PC surface (θ = 0º). Where $\boldsymbol{B_0}$ and $\boldsymbol{B_1}$ are defined as,

$$\begin{cases} \boldsymbol{B_0} = \frac{1}{2}\begin{bmatrix} (1+\xi_{01}) & (1-\xi_{01}) \\ (1-\xi_{01}) & (1+\xi_{01}) \end{bmatrix}, \\ \boldsymbol{B_1} = \frac{1}{2}\begin{bmatrix} (1+\xi_{12})e^{-ik_{1z}d_s} & (1-\xi_{12})e^{ik_{1z}d_s} \\ (1-\xi_{12})e^{-ik_{1z}d_s} & (1+\xi_{12})e^{ik_{1z}d_s} \end{bmatrix}. \end{cases} \tag{11}$$

Where $\xi_{01} = \frac{n_0}{n_1}$ and $\xi_{12} = \frac{n_1}{n_2}$ are the ratios of refractive indices in interfaces between different regions of materials; $k_{1z} = \frac{2\pi n_1}{\lambda_0}$ is the z-component of the wavenumber in the slab (λ_0 is the resonance wavelength in free space). Therefore, the FP reflection can be solved from Equations (9), (10) and (11) as,

$$R_f = |r_f|^2 = \left|\frac{b_{21}}{b_{11}}\right|^2 \tag{12}$$

where r_f is the continuum reflected amplitude from the vertical FP cavity. The FP reflection can be chosen to be incorporated or not with the guided resonance reflection (determined by the guided resonance at the FP reflection maximum or minimum) depending on the applications. In this work, the FP reflection is

isolated from the guided resonance reflection because this study focuses on the effect of the guided resonance reflection itself.

S-1.4 Reflection from the photonic crystal surface

The reflection from the PC surface demonstrates a Fano resonance mode whose characteristic is determined by interference between the vertical FP cavity reflection and the guided resonance mode, and can be approximated as

$$R_p = |r_p|^2 = \left| r_f + F_f \frac{\gamma}{i(\omega - \omega_0) + \gamma} \right|^2 \quad (13)$$

Where r_p is the overall reflected amplitude from the PC surface; r_f is the continuum direct reflected amplitude from the vertical FP cavity reflection in a complex format (for simplicity, r_f can be approximated with a second order polynomial fitting as $r_f(\omega) = a_0 + a_1\omega + a_2\omega^2$ within certain frequency range, where a_0, a_1 and a_2 are fitting coefficients[64-67,77]); ω_0 and γ are the resonance angular frequency and bandwidth of the guided resonance mode (with Lorentzian lineshape); F_f is a complex factor of Fano resonance for the guided resonance mode and $F_f = be^{-i\varphi}$ (where b is the amplitude for guided resonance mode; φ is the phase difference between the FP reflection and the guided resonance mode). Along the axis of angular frequency, the overall reflection from the PC surface can be categorized into the following two regions: (a) around resonance frequency of the guided resonance mode ($|\omega - \omega_0| \leq \gamma$), the momentum-matching condition is satisfied and hence the reflection demonstrates a Fano resonance with asymmetric lineshape, due to interference between the FP cavity and the guided resonance mode (the resonance spectrum achieves a symmetric lineshape when $\varphi = 0$ or $r_f = 0$); (b) far from the resonance frequency of the guided resonance mode ($|\omega - \omega_0| >> \gamma$), the continuum FP reflection becomes the dominant response, since the momentum-matching condition is not satisfied in these regions and thus the incident wave can no longer be coupled into the guided resonance mode.

S-2 The properties of PC

S-2.1 Quality factor (Q)

The Quality factor (Q-factor) characterizes the leaky property of the PC and is defined as the ratio of the energy stored in the resonator to the power loss. Here the Q-factor is estimated as

$$Q = \frac{\lambda_0}{\Delta\lambda} \quad (15)$$

where λ_0 is the resonance wavelength; $\Delta\lambda$ is width of the resonance mode and can be measured at the full width at half maximum (FWHM) of the mode. The Q-factor is estimated as ~160 for the PC surface in this work and it can vary if angle θ_i of the incident radiation varies .The magnitude of the electromagnetic intensity localized on the PC is proportional to the Q-factor of the resonance, which is determined by varying the parameters of the nano structure.

S-2.2 Physical mechanism of peak intensity shift (PIS)

It is essential to understand the physical meaning of the peak intensity shift measured on PC surface. In theory, there are several potential mechanisms, such as absorption and scattering that can cause a variation in the peak intensity value of the reflection spectrum. The photons in the leaky mode coupled

into the cellular materials on the PC surface can be approximated as a plane wave in a small local volume (e.g., in a voxel with a volume of $\delta V = 0.6\times0.6\times0.06\ \mu m^3$),

$$|E(\boldsymbol{r},t)|^2 = \left|E_0 e^{i(\bar{k}r-\omega t)}\right|^2 = \left|E_0 e^{i\left(\frac{2\pi(n+i\kappa)r}{\lambda_0}-\omega t\right)}\right|^2 = \left|e^{-\frac{2\pi\kappa r}{\lambda_0}} E_0 e^{i\left(\frac{2\pi n r}{\lambda_0}-\omega t\right)}\right|^2 = |e^{-\alpha}|^2\left|E_0 e^{i(kr-\omega t)}\right|^2. \quad (16)$$

Where $\boldsymbol{r}$ is the spatial coordinate vector and t is time; $\bar{k} = 2\pi\bar{n}/\lambda_0 = 2\pi(n+i\kappa)/\lambda_0$ is the complex wavenumber. For the complex refractive index $\bar{n} = n + i\kappa$, its real part n is the refractive index and indicates the phase velocity of the propagating wave. A change in the real part (n) of the complex refractive index can cause the peak wavelength shift (PWS) in the reflection spectrum when satisfying a new momentum-matching condition (as shown in simulation results in Fig. 2e-g). It is worthy to note that the peak intensity value (PIV) does not show obvious reduction around the resonance wavelength if only increasing the refractive index of the background medium in the FDTD simulation (Fig. 2e-f) (although it is difficult to find a dielectric material without finite molecule sizes to demonstrate this phenomenon in reality). The imaginary part (κ) of the complex refractive index is the extinction coefficient and indicates attenuation in reflection due to absorption as the wave propagating through the material. If the extinction coefficient of the material above PC slab is nonzero (e.g. a gold nanoparticle) in the target wavelength range (around λ_0 = 626 nm in this work), the absorption of the electromagnetic radiation can happen around the target wavelength range and the measured PIV can get attenuated by a factor $\mu_a = |e^{-\alpha}|^2$.

Another attenuation mechanism is the scattering of light around the concentrated loci of cellular molecules atop the PC surface. Scattering is a process through which some of the incident light is deviated from its original trajectory due to the non-uniformity of the local materials. On the PC surface, some of the scattered light (with one or more optical paths) from the cellular molecules couples back to the guided resonance mode with a shifted resonance wavelength ($\lambda+\Delta\lambda$), then couples out again to the leaky modes under the PC slab, and finally is measured by the PROM imaging system as the reflection spectrum. During this light-matter coupling and scattering process, only the backscattered light is collected and all of the light scattered to other directions is lost. Comparing before the cellular molecules are present, there is a peak intensity shift after the cellular molecules attach to the PC surface. The larger the scattering object (when the refractive index of the object remains the same), the more light is removed from the light path that leads back to the microscope objective lens, and hence the more the measured peak reflected intensity is reduced. This effect can be demonstrated in FDTD simulation by varying the size of a low refractive index contrast spherical dielectric particle on the PC surface (Fig. 2h-j).

In this work, the peak intensity shift is the overall extinction effect of both absorption and scattering. However, it is known that the cellular materials typically have strong absorption in the range of near infrared wavelengths (700-1000 nm) rather than in visible spectrum used for PROM (400-700 nm), and hence the contribution from absorption can be assumed to be negligible. Therefore, scattering is assumed to be the dominant effect in the cellular molecules in PROM imaging in this work.
